# Considerations for expanding community exercise programs incorporating a healthcare-recreation partnership for people with balance and mobility limitations: a mixed methods evaluation

**DOI:** 10.1186/s13104-018-3313-x

**Published:** 2018-04-02

**Authors:** Nancy M. Salbach, Jo-Anne Howe, Diem Baldry, Saira Merali, Sarah E. P. Munce

**Affiliations:** 1 Department of Physical Therapy, University of Toronto, 160-500 University Avenue, Toronto, ON M5G 1V7 Canada; 2 University Health Network-Toronto Rehabilitation Institute, 550 University Avenue, Toronto, ON M5G 2A2 Canada

**Keywords:** Community, Task-oriented exercise, Balance, Mobility, Spread, Scale-up

## Abstract

**Objective:**

To increase access to safe and appropriate exercise for people with balance and mobility limitations, community organizations have partnered with healthcare providers to deliver an evidence-based, task-oriented group exercise program in community centers in Canada. We aimed to understand challenges and solutions to implementing this program model to inform plans for expansion.

**Results:**

At a 1-day meeting, 53 stakeholders (healthcare/recreation personnel, program participants/caregivers, researchers) identified challenges to program implementation that were captured by seven themes: Resources to deliver the exercise class (e.g., difficulty finding instructors with the skills to work with people with mobility limitations); Program marketing (e.g., to foster healthcare referrals); Transportation (e.g., particularly from rural areas); Program access (e.g., program full); Maintaining program integrity; Sustaining partnerships (i.e., with healthcare partners); and Funding (e.g., to deliver program or register). Stakeholders prioritized solutions to form an action plan. A survey of individuals supervising 28 programs revealed that people with stroke, acquired brain injury, multiple sclerosis, and Parkinson’s disease register at 95–100% of centers. The most prevalent issues with program fidelity across centers were not requiring a minimum level of walking ability (32%), class sizes exceeding 12 (21%), and instructor-to-participant ratios exceeding 1:4 (19%). Findings provide considerations for program expansion.

**Electronic supplementary material:**

The online version of this article (10.1186/s13104-018-3313-x) contains supplementary material, which is available to authorized users.

## Introduction

Many chronic health conditions, such as stroke and multiple sclerosis, result in persistent balance and mobility limitations [1–3]. Balance and mobility limitations contribute to functional dependence [1] and physical inactivity [4] which can further diminish health [1, 5–7]. Community-based exercise programs (CBEPs) that involve a healthcare professional have emerged in the United Kingdom [8–13], Australia [14], Italy [15], Canada [16], and the United States [17]. These programs can facilitate safe exercise participation for people with disabilities to help mitigate the negative consequences of balance and mobility limitations [12–16, 18].

In Canada, a group, task-oriented, CBEP incorporating a healthcare-recreation partnership (CBEP-HRP) called “Together in Movement and Exercise” (TIME™) has been developed [16, 19]. This program has been proven safe and appropriate for people with balance and mobility limitations who can walk at least 10 m independently and have sufficient cognitive and communication ability to function in a group setting [16]. In the TIME™ partnership, healthcare professionals, typically physical therapists, train and support fitness instructors to deliver the exercise program in community centers run by recreation organizations. The partnership was designed to maintain program quality and safety and support participant referral.

TIME™ involves a 1-h exercise class provided twice a week for 12 weeks. Classes involve seated warm-up and cool-down exercises, and practice of functional exercises (e.g., sit-to-stand, modified lunges, step-ups, walking), with standardized progressions, designed to improve balance and mobility. A minimum instructor-plus-volunteer-to-participant ratio of 1:4 is required to maintain adequate supervision and exercise progression [16]. Family members are invited to assist during the class if needed.

After a pilot study demonstrated the safety, feasibility, and potential benefit of the TIME™ model [16], a toolkit [20] that includes exercise guidelines and space/equipment requirements to run the program was developed. Using this toolkit, coordinators within stroke networks and regional health authorities facilitated spread of the TIME™ program to 28 community centers in Ontario and British Columbia, Canada by 2014. Although the ultimate goal of the TIME™ model was to enable long-term access to safe and beneficial exercise for people with balance and mobility limitations, the extent to which the TIME™ program was being delivered as designed, and the feasibility of sustaining the program were unclear. Thus, the aim of this study was to identify challenges with initial and sustained implementation of the TIME™ program model and solutions as perceived by program stakeholders. Results are expected to inform action plans to improve access to group, task-oriented, CBEP-HRPs for people with balance and mobility limitations.

## Main text

### Methods

A 1-day stakeholder meeting and two follow-up surveys were undertaken. Seventy-seven individuals from academic, healthcare, and recreation sectors from across Canada who had experience with the TIME™ program or a similar program were invited to participate in the stakeholder meeting in May 2014. Recreation coordinators obtained permission from TIME™ exercise participants and caregivers to contact them with an invitation to participate.

Prior to the meeting, individuals were asked to document challenges, facilitators and strategies to implementing or participating in CBEPs using a standardized form (Additional file 1). Forms were submitted at meeting registration. Data were synthesized and presented during the meeting (agenda in Additional file 2). Morning meeting activities involved sharing of experiences with delivering or participating in the TIME™ program, research evidence supporting group, task-oriented training, and funding and policy issues affecting program expansion. In the afternoon, participants, seated by stakeholder group, were asked to identify and report on the two most important challenges with implementing the TIME™ model. Meeting facilitators (authors NMS & DB) documented the challenges. Each participant was then asked to vote for his/her top two challenges using a ballot that was color-coded by stakeholder group. After collecting the ballots, each stakeholder group was assigned one challenge and asked to identify and report on strategies to address the challenge. The strategies were documented. Immediately following the meeting, participants were invited to complete an online questionnaire to rate the level of priority of strategies as: not a priority, low priority, medium priority, and high priority. In September 2014, supervisors of TIME™ programs at 28 community centers were invited to complete an online questionnaire (Additional file 3) designed to characterize TIME™ program delivery.

Frequencies and percentages were used to summarize meeting and survey data. A descriptive content analysis [21] of the qualitative data from pre-meeting and meeting activities describing challenges to program implementation was performed. Similar challenges were clustered to identify themes.

### Results

Of the 77 individuals invited, 53 (69%) attended the meeting. Of the 53 attendees (positions and organizations are listed in Additional file 4), 21 (40%) completed the pre-meeting activity, 40 (75%) participated in discussions at stakeholder-specific tables of 6 stakeholder groups to identify challenges and solutions related to TIME™ program delivery, and 42 (79%) rated the priority level of solutions post-meeting. Stakeholders who discussed program delivery challenges and solutions included 7 healthcare professionals, 9 healthcare system representatives, 11 fitness instructors, 9 recreation coordinators/managers, 3 researchers and 1 exercise participant.

Challenges identified during meeting discussions and voting results are described in Additional file 5. Challenges were captured by seven themes. (1) *Resources to deliver the exercise class*: Recreation centers faced issues related to inadequate space to run the class and store equipment and inappropriate exercise equipment. Recreation staff described difficulty finding instructors with the skills to work with people with multiple health conditions, language barriers, and low mobility levels, and to adapt the exercises to account for changes in participant ability or injury. Some centers were faced with high staff turnover; thus, maintaining a roster of trained staff over time was difficult. Recruiting, training and scheduling volunteers who were sometimes needed to maintain the 1:4 instructor-plus-volunteer-to-participant ratio was also noted as challenging. (2) *Program marketing*: Healthcare and recreation personnel recognised the challenge of promoting and raising awareness of the program among healthcare and rehabilitation professionals who could endorse the program and support referral to ensure adequate registration. (3) *Transportation*: Exercise participants and healthcare/recreation personnel agreed that transportation to the program could be costly and inconvenient. Adapted transport services did not consistently arrive on schedule, were cancelled during inclement weather, or were unavailable in rural areas. (4) *Program access*: Registration was not always possible. The program was either full or the exercises were inappropriate for some clients with multi-morbidities and low mobility levels and some clients with high functional levels who had already taken the program. These challenges were perceived as preventing long-term exercise participation. (5) *Maintaining program integrity*: This challenge related to ensuring consistent delivery of the program as intended over time across centers. (6) *Sustaining partnerships*: Maintaining roles, communication and collaboration between healthcare and recreation partners was considered challenging. (7) *Funding*: All stakeholders identified the need for additional funding to sustain the TIME™ program model. Recreation partners needed funding for staff wages, equipment, and program expansion; healthcare providers required funding to offer training and support; and clients needed funding to pay for program registration and transportation. Table 1 lists 29 strategies proposed to address the program challenges and associated priority ratings.

**Table 1:** Prioritization of strategies targeting challenges to implementing the TIME™ model for people with balance and mobility limitations (n = 42)^a^

Challenge	Strategy	n	Priority rating n (%)			
			Not a priority	Low	Medium	High^a^
1. Insufficient funding for recreation providers to run the exercise program and for healthcare providers to offer training and support	1. Submit a proposal to your regional health authority (e.g., Local Health Integration Network) to fund exercise programs in the region	42	0 (0)	2 (5)	9 (21)	*31 (74)*
	2. Make the case to hospital managers to fund physical therapists to partner with recreation providers to deliver CBEPs as an investment in public health	42	2 (5)	5 (12)	14 (33)	21 (50)
	3. Leverage existing resources of the Canadian Stroke Strategy (e.g., Provincial coordinators could educate hospital staff to refer patients to exercise programs)	40	1 (3)	5 (13)	14 (35)	20 (50)
	4. Approach condition-specific charities (e.g., MS Society, Heart & Stroke Foundation, etc.)	42	1 (2)	9 (21)	16 (38)	16 (38)
2. Maintenance of program integrity: this refers to delivering the exercise program as intended both at start up and over time	1. Consistent use of training materials (e.g., slides in toolkit for instructor training and task-related exercise program guidelines	42	0 (0)	1 (2)	11 (26)	*30 (71)*
	2. Exercise program/facility certification (e.g., programs need to meet safety/quality criteria similar to Heart Wise certification)	42	1 (2)	2 (5)	15 (36)	24 (57)
	3. Funding for a healthcare position in the community to refer patients to exercise programs and collaborate with exercise providers	42	2 (5)	4 (10)	15 (36)	21 (50)
	4. Physical therapist visits to exercise programs to consult with fitness instructors at recreation centers	42	0 (0)	3 (7)	20 (48)	19 (45)
3. Sustainability of exercise programs: this refers to the continued provision of CBEPs over time	1. Ongoing inter-professional communication/collaboration between rehabilitation and recreation providers	41	0 (0)	0 (0)	5 (12)	*36 (88)*
	2. Availability of ongoing training of new fitness instructors	42	0 (0)	1 (2)	11 (26)	*30 (71)*
	3. Canadian stroke system representatives in each province advocate for exercise programs across regions	42	0 (0)	2 (5)	17 (40)	23 (55)
	4. Canadian stroke system representatives in each province help patients overcome barriers to exercise participation to enable access to exercise programs across regions	42	0 (0)	7 (17)	17 (40)	18 (43)
4. Marketing of the program	1. Links with physicians and healthcare providers	42	0 (0)	1 (2)	13 (31)	*28 (67)*
	2. Links with key stakeholder groups such as peer support groups (e.g., stroke support groups), condition-specific groups (e.g., MS Society, Heart & Stroke Foundation), and homecare services (e.g., Community Care Access Centers)	42	0 (0)	1 (2)	14 (33)	*27 (64)*
	3. Standardised marketing materials (e.g., videos, pamphlet, community of practice)	42	0 (0)	3 (7)	13 (31)	*26 (62)*
	4. Links with key systems (e.g., Healthline (a website that lists healthcare and community services in Ontario), TIME™ website)	42	0 (0)	4 (10)	14 (33)	24 (57)
5. Staff training: refers to the challenge of training instructors to have the multiple skills required to deliver these exercise programs (e.g., adapting exercise difficulty to account for participant ability or injury)	1. Consulting with key people as problems arise (e.g., TIME™ educators)	41	0 (0)	1 (2)	12 (29)	*28 (68)*
	2. Regular meetings of fitness instructors across sites to share issues and problem solve (e.g., Skype, conference call)	42	0 (0)	5 (12)	19 (45)	18 (43)
	3. Webinars for educational opportunities	42	0 (0)	5 (12)	20 (48)	17 (40)
	4. Online discussion forum (e.g., social media)	42	0 (0)	9 (21)	19 (45)	14 (33)
6. No access to recruit exercise participants directly from rehabilitation hospital programs	1. Form links between rehabilitation and recreation providers (network meetings and promotional visits between community-based exercise providers and rehabilitation clinics)	42	1 (2)	0 (0)	4 (10)	*37 (88)*
	2. Bridging with other community based programs (e.g., joint advertisement/accreditation for Heart Wise and TIME™)	41	0 (0)	3 (7)	18 (44)	20 (49)
	3. Marketing through newspaper, magazines, pamphlet distribution	41	0 (0)	11 (27)	12 (29)	18 (44)
	4. Advertisement targeted at adult living communities	42	1 (2)	7 (17)	22 (52)	12 (29)
	5. Online forum: e.g., municipal recreation fitness/supervisor and instructors across provinces for Q & A	42	0 (0)	7 (17)	25 (60)	10 (24)
7. Exercise program full and not open to new registrants: refers to when exercise participants re-register and there are no or few spaces in the class for new registrants	1. Offer additional programs at same or other locations	42	0 (0)	3 (7)	10 (24)	*29 (69)*
	2. Offer maintenance program at various levels for graduates and people with more severe deficits	42	0 (0)	3 (7)	13 (31)	*26 (62)*
	3. Educate exercise participants about other available programs offered at the facility (know options)	42	0 (0)	2 (5)	18 (43)	22 (52)
	4. Where space is the issue, network with other organizations or providers to find space to launch more programs	42	0 (0)	1 (2)	20 (48)	21 (50)

^a^ 42 individuals included healthcare professionals (33%), fitness instructors (33%), recreation coordinators/managers (38%), and researchers (10%)

^b^ Italic typeface indicates a strategy rated as a high priority by ≥ 60% of survey respondents

Seventeen supervisors of TIME™ programs run by 25 organizations in 28 community centers completed the online questionnaire (100% response rate). Across 28 centers, TIME™ programs had been running for ≤ 1 year (14%), 1–2 years (46%), 2–4 years (32%), and 6–8 years (7%). Exercises were performed in a circuit (original version) or three superstations (three exercises/superstation; updated version), in 57 and 29% of centers, respectively. Most frequently, classes were 60 min in length (89%), provided twice a week (57%) for 12 weeks (36%), and 3 times per year (39%). Volunteers and caregivers were permitted to assist in 75 and 89% of centers, respectively. Table 2 describes characteristics of program referral, advertisement, intake, format, and registration.

**Table 2:** Characteristics of TIME™ programs at 28 community centers

Program characteristic	No. responding^a^	n (%)
Referral and advertisement		
Referral by at least 1 hospital-based healthcare professional	23/25	23 (100)
Multi-program brochure	24/25	24 (100)
Website	25/25	24 (96)
Program-specific brochure	24/25	21 (88)
Free sessions offered to orient interested individuals	23/25	20 (87)
Charitable organizations	23/25	19 (83)
Other (e.g., advertising in local homecare and physical therapy clinics, newspapers; local TV station interview; visiting doctors’ offices/hospitals)	8/25	4 (50)
Admission criteria		
Able to walk 10 m independently ± an assistive device	25/25	17 (68)
Self-reported balance or mobility limitation	25/25	17 (68)
Medical clearance form signed by physician or other provider	24/25	16 (67)
Other (e.g., PAR-Q+, medication form and waiver; no criteria)	23/25	3 (13)
Criterion to exclude based on high ability level		
Able to walk 30 min continuously	23/25	7 (30)
No criteria	23/25	5 (22)
Other (e.g., ability to perform exercises easily in first class)	23/25	5 (22)
Conditions causing balance/mobility limitations in registrants		
Stroke	25/25	25 (100)
Acquired brain injury	24/25	24 (100)
Multiple sclerosis	20/25	20 (100)
Parkinson’s disease	19/25	18 (95)
Other (e.g., spinal cord injury, arthritis, frail elderly, cancer, and vertigo)	19/25	19 (95)
Typical number of participants per class	28/28	
0–4		8 (29)
5–8		8 (29)
9–12		12 (43)
Maximum number of participants permitted per class	28/28	
6–9		13 (46)
10–12		9 (32)
13–16		6 (21)
Minimum number of registrants to run a class	28/28	
2–4		22 (79)
5–8		4 (14)
9–11		2 (7)
Typical number of instructors per class	28/28	
1 instructor per class		5 (18)
2 instructors per class		19 (68)
3 instructors per class		1 (4)
Other [e.g., adding 1 instructor if class size > 6 (n = 2); 8–10 volunteers (n = 1)]		5 (18)
Typical number of volunteers per class	28/28	
0 volunteers per class		9 (32)
1 volunteer per class		10 (36)
2 volunteers per class		3 (11)
≥ 3 volunteers per class		6 (21)
Typical instructor + volunteer-to-participant ratio	27/28	
≤ 1:4		22 (81)
> 1:4 (includes one center that reported 1:4–5)		5 (19)
Typical number of caregivers per class	28/28	
0 caregivers per class		4 (14)
1 caregiver per class		8 (29)
2 caregivers per class		10 (36)
Variable number, unable to specify.		6 (21)
Percentage of TIME™ participants that typically re-register (%)	28/28	
0		1 (4)
1–25		10 (36)
26–50		2 (7)
51–75		4 (14)
76–100		11 (39)
Percentage of TIME™ participants typically unable to re-register as class is full (%)	26/28	
0		6 (23)
1–25		19 (73)
26–50		0 (0)
51–75		0 (0)
76–100		1 (4)
TIME™ program has a waiting list^b^	28/28	11 (39)
Percentage of TIME™ participants that typically register for other exercise classes at the community center (%)	28/28	
0		5 (18)
1–25		17 (61)
26–50		3 (11)
51–75		0 (0)
76–100		3 (11)
Exercise programs that TIME™ participants register for	23/28	
Pool classes		20 (87)
Yoga or chair yoga		9 (39)
Weight room programs		8 (35)
Gentle fit or seated fitness classes		6 (26)
Individual physical activity sessions		3 (13)
Tai chi		1 (4)

^a^ Denominator refers to either 25 organizations or 28 community centers

^b^ Respondents reported having 5, 6, and 9 people on a waiting list for the TIME™ program

### Discussion

This mixed methods study revealed a range of program challenges related to recreation center resources, program marketing, transportation, access, integrity, funding, and sustaining partnerships, relevant to six stakeholder groups. Stakeholders identified high priority strategies targeting each of these challenges, with the exception of transportation. These strategies can be used to inform the development of action plans to help implement and sustain the TIME™ program. Following spread of the TIME™ program to 28 community centers, certain recommended program elements, including class format and duration, participation of caregivers, involvement of and referral of participants by healthcare professionals, were maintained, while others, such as admission criteria, weekly class frequency, program duration, maximum class size, instructor-to-participant ratio, and use of volunteers, were adapted.

Some challenges to delivering the TIME™ program, such as program cost and transportation, have been noted previously by people with stroke [22–24], HIV [25], and COPD [26, 27], as primary barriers to participation in structured exercise programs. Individuals in these studies recommended making CBEP-HRPs widely available [24, 26]. The ability to attend programs in close proximity to one’s home was perceived to minimize travel time and cost of transportation, and offset the negative impact of unreliable public transit, and inclement weather on program attendance [24, 26]. Subsidization of program cost was desired [26] as people with physical disability may be receiving a fixed income [25, 26]. Results from the current study further highlight the need for financial support of healthcare and recreation partners to sustain the CBEP-HRP model. The issue of program funding was recently investigated in a survey of providers of 14 exercise program programs for people with stroke in Scotland [28]. In this survey [28], three programs run by physiotherapists, nurses and assistants in healthcare settings to help transition people from hospital to independent exercise, were government-funded. Although participation was free, only one 10-week session was provided which may be insufficient to facilitate lifelong participation in physical activity. The strategy proposed in the current study to obtain regional healthcare funding for programs like TIME™ would provide people with physical disability with ongoing opportunities to exercise.

The importance of maintaining partnerships to sustain program referral, delivery, and integrity was underscored in our study. People with physical disability prefer a trusted healthcare practitioner to refer them to CBEPs, as this reassures them that the program is safe and appropriate [27]. Knowledge that a healthcare professional has continued involvement in a CBEP, as in the TIME™ program, provides further reassurance [29]. As proposed in the current study, standardized marketing materials used by a local facilitator may help foster partnerships with physicians, charities, peer support groups, and homecare service providers to help support program registration. Finally, opportunities for instructor training and continuing education, and the continued involvement of a healthcare provider in program delivery through periodic visits, may help to minimize local program adaptations that could decrease program quality and safety. For example, a third of centers in the current study did not apply the admission criteria of ability to walk 10 meters independently with or without an assistive device, considered a core program element [16]. This criterion helps to ensure participants have a minimum level of mobility to safely perform and benefit from the program exercises. Similarly, approximately 20% of centers reported a maximum class size of 14–16, and an instructor-to-participant ratio exceeding 1:4. These practices may reflect the inclusion of individuals with a higher level of balance and mobility ability that do not require close supervision. However, a ratio of 1:4 is important to ensure adequate supervision and participant safety. Future research should aim to better understand the role of healthcare providers in maintaining the safety and quality of CBEP-HRPs. Finally, CBEP-HRPs for individuals with more severe balance and mobility limitations as well as a process for graduating TIME™ program participants to more advanced exercise programs, were suggested to address wait lists observed in 39% of community centres offering the TIME™ program and enable exercise participation for a larger group of individuals.

## Conclusions

Stakeholders involved in the unplanned spread of the CBEP-HRP TIME™ model in a publicly-funded healthcare system encounter challenges related to inadequate funding and infrastructure that may threaten the sustainability of these programs. Local application of the solutions proposed in this research is likely to result in slow and haphazard improvements as it will depend on the resources of individual organizations. Public health agencies, supported by a mandate and dedicated funding, will find our study findings relevant to planning for systematic development and scale-up of CBEP-HRPs to enable widespread and equitable access to exercise participation for people with a wide range of balance and mobility limitations.

## Limitations

Challenges and strategies identified in this study may primarily reflect the priorities of healthcare and recreation professionals as they had a high degree of representation. Their opinions, however, were informed by presentations made by exercise participants and caregivers early in the meeting. Seating participants by stakeholder group and inclusion of anonymous voting were strengths of the meeting process that helped to ensure representation of multiple stakeholder perspectives.

## Additional files

**Additional file 1.** Pre-meeting Activity.

**Additional file 2.** Meeting Agenda.

**Additional file 3.** TIME^TM^ Survey Questionnaire.

**Additional file 4.** Meeting participant positions and organizations.

**Additional file 5.** Identification and endorsement of challenges.

## Abbreviations

CBEP: community-based exercise program
CBEP-HRP: community-based exercise program incorporating a healthcare-recreation partnership
TIME™: Together in Movement and Exercise
