# Supplementary material for: Considerations for expanding community exercise programs incorporating a healthcare-recreation partnership for people with balance and mobility limitations: a mixed methods evaluation
Source: BMC Res Notes. 2018 Apr 2;11:214. doi: 10.1186/s13104-018-3313-x (PMC5879753; doi:10.1186/s13104-018-3313-x)
Supplement: Supplementary file 1 — Additional file 1. Pre-meeting Activity. [file 13104_2018_3313_MOESM1_ESM.pdf]

Additional File 1
PRE-MEETING ACTIVITY

Please check the appropriate box to indicate which of the following positions best applies to you:

- ☐ **Fitness instructor**
- ☐ **Recreation manager/supervisor**
- ☐ **Healthcare professional**
- ☐ **TIME^{TM} participant**
- ☐ **TIME^{TM} caregiver**
- ☐ **Researcher**
- ☐ **Other:** ______________________________

Instructions: Reflect on your experience being involved with community exercise programs for people with balance and mobility limitations like the TIME^{TM} program. Think about the challenges you experienced with starting up, expanding and running the program over time. In the tables below, please write down some of the challenges you experienced, facilitators that made things easier, and strategies you implemented to overcome the challenges. Examples of *Challenges*, *Facilitators* and *Strategies* are provided below. In the table, please state examples from your own experiences.

There are three tables for the phases of 1) starting up, 2) expanding and 3) sustaining the exercise program over time. Enter your comments related to each of these phases in the appropriate table. You do not need to complete a table if you don't have experience with that phase of exercise program delivery.

Note: If you are an exercise participant or caregiver, enter comments about your experience in the program for the first time (starting up table) and over time (sustaining table).

By "Challenges" we mean a barrier or obstacle to achieving the desired goal. Barriers to delivering an exercise program might relate to:

- people (e.g., knowledge, skill, attitude);
- the exercise program (e.g., amount and cost of equipment, need for space, need for human resources, complexity of exercises, need for expertise, liability, cost);
- organization (e.g., vision, mission, culture).

By "Facilitators" we mean resources (physical or human), or individual characteristics that currently exist and that help to achieve the desired goal. For example, you may already employ a fitness instructor with experience working with people with physical disability.

By "Strategies" we mean a planned action you undertook to address a particular challenge, such as organizing a workshop to educate and train fitness instructors to deliver the exercise program.

Everyone's comments will be used anonymously to make a comprehensive list of Challenges, Facilitators and Strategies at the Meeting.

STARTING UP AN EXERCISE PROGRAM for people with balance and mobility limitations
Starting Up **refers to initiating a program for the first time (no prior experience)**

CHALLENGES	FACILITATORS	STRATEGIES

EXPANDING AN EXERCISE PROGRAM WITHIN AN ORGANISATION for people with balance and mobility limitations<br>***Expanding*** **refers to adding programs at the same site or at a different site (ie community centre) within an organization that is already running the program**		
**CHALLENGES**	**FACILITATORS**	**STRATEGIES**

SUSTAINING AN EXERCISE PROGRAM for people with balance and mobility limitations
***Sustaining* refers to continuing to offer the program over time (eg years)**

CHALLENGES	FACILITATORS	STRATEGIES
