# Supplementary material for: Considerations for expanding community exercise programs incorporating a healthcare-recreation partnership for people with balance and mobility limitations: a mixed methods evaluation
Source: BMC Res Notes. 2018 Apr 2;11:214. doi: 10.1186/s13104-018-3313-x (PMC5879753; doi:10.1186/s13104-018-3313-x)
Supplement: Supplementary file 2 — Additional file 2. Meeting Agenda. [file 13104_2018_3313_MOESM2_ESM.pdf]

Additional File 2

Meeting Agenda

**8:30-9:00 am**	**Registration and Breakfast**
**9:00-9:15 am**	**Welcome and Introductions**<br><br>*Diem Baldry, Nancy Salbach, Jo-Anne Howe*
**9:15-9:35 am**	**Conceptual foundations for the implementation and expansion of group, task-oriented exercise programs**<br><br>*Nancy Salbach*
**9:35-10:20 am**	**Experiences implementing task-oriented exercise programs targeting balance and mobility: Benefits, challenges, and strategies for improvement**<br><br>*Large group discussion of providers of the Together In Movement and Exercise (TIME^{TM}) and similar programs (Facilitator: Diem Baldry)*
**10:20-10:35 am**	**NUTRITION BREAK**
**10:35-11:05 am**	**Uptake of the TIME^{TM} program by the British Columbia Fraser Health Authority: Implications for other provinces**<br><br>*Guest speakers: Jason Moller and Joy Parsons*
**11:05-11:35 am**	**Experiences and challenges seeking out and participating in exercise programs**<br><br>*TIME^{TM} participants and caregivers (Facilitator: Diem Baldry)*
**11:35-12:30 pm**	**Role of healthcare policy and funding in the implementation and expansion of task-oriented exercise programs**<br><br>*Expert Panel: Dr. Mark Bayley and Dr. Samir Sinha*<br>*(Moderator: Nancy Salbach)*
**12:30-1:30 pm**	**LUNCH & NETWORKING**
**1:30-1:45 pm**	**Role of education and training in implementing and sustaining a community-based exercise program**<br><br>*Guest speaker: Alda Tee*
**1:45-1:55 pm**	**Challenges implementing and expanding task-oriented exercise programs: A comprehensive overview**

	Nancy Salbach and Saira Merali
**1:55-2:35 pm**	**Which challenges should be addressed first?** ***Sharing your perspectives*** **(Small group discussion)**<br><br>*Diem Baldry*
**2:35-2:40 pm**	**ACTIVE BREAK**
**2:40-2:50 pm**	**Which challenges should be addressed first?** ***Casting your vote***<br><br>*Diem Baldry*
**2:50-3:05 pm**	**Overcoming the challenges of implementing and expanding task-oriented exercise programs: Facilitators and strategies**<br><br>*Nancy Salbach*
**3:05-3:20 pm**	**NUTRITION BREAK**
**3:20-3:55 pm**	**Which strategies are key to overcoming the challenges?** ***Sharing your perspectives*** **(Small group discussion)**<br><br>*Diem Baldry*
**3:55-4:05 pm**	**Which strategies are key to overcoming the challenges?** ***Casting your vote***<br><br>*Diem Baldry*
**4:05-4:25 pm**	**Where do we go from here?**<br><br>*Diem Baldry and Nancy Salbach*
**4:25-4:30 pm**	**Summary and Closing Remarks**<br><br>*Nancy Salbach*
