# Supplementary material for: Considerations for expanding community exercise programs incorporating a healthcare-recreation partnership for people with balance and mobility limitations: a mixed methods evaluation
Source: BMC Res Notes. 2018 Apr 2;11:214. doi: 10.1186/s13104-018-3313-x (PMC5879753; doi:10.1186/s13104-018-3313-x)
Supplement: Supplementary file 3 — Additional file 3. TIMETM Survey Questionnaire. [file 13104_2018_3313_MOESM3_ESM.pdf]

Additional File 3

TIME™ Survey Questionnaire

Introduction

We are conducting a survey of TIME™ providers to obtain information on how different centres advertise and deliver the TIME™ program. We are collecting this information because some of you have indicated you would like to know how other centres deliver their programs. Our plan is to summarize the information we collect to share with you and to present in a manuscript for publication that will help to raise awareness of the TIME™ programs and the organisations that deliver it.

The information that we are collecting will be kept confidential. In the summary, information will be presented using summary statistics such as counts and percentages or ranges. Information will not be linked to a specific program. If you have any questions, feel free to contact Nancy Salbach at nancy.salbach@utoronto.ca.

If the TIME™ program is delivered **in the same way** at multiple sites within your organization, then **one person from your organization** can complete the survey questionnaire for all of the sites.

1. Please indicate the community centres for which you are completing the survey questionnaire - Check all that apply (response options removed to maintain confidentiality).

2. Please list the month and year (e.g., Jan 2010) when your centre started offering the TIME™ program. If you are completing the questionnaire for multiple sites, list the site, then the start date (eg Centre name: Jan 2010). _________________________

Comments: _________________________

TIME™ Program Advertisement

Please indicate whether your organization uses each of the following strategies to advertise the TIME™ program:

	**Yes**	**No**
3. Program is described on the organization's website	●	○
4. TIME™ specific flyer/brochure is available at the centre	○	○
5. TIME™ program is listed in a multi-program flyer/brochure that is available at the centre	○	○
6. You have an established relationship with one or more hospital-based healthcare professionals who refer patients	○	○
7. You provide free sessions to orient interested individuals to the centre	○	○

	Yes	No
8. Charitable organizations advertise the program to their members	○	○
9. Other:	○	○

If Other, please specify: ______________________________

TIME™ Program Intake

Which of the following criteria must be satisfied for an individual to participate in your TIME™ program?

	Yes	No
10. Person describes having a balance or mobility limitation	○	○
11. Person is able to walk 10 metres independently with or without an assistive device	○	○
12. Person must submit a medical clearance form signed by their physician or other healthcare provider	○	○
13. Do you have additional admission criteria?	○	○

If Yes, please specify: ______________________________

14. What criteria do you use to classify someone as “too high level” for the TIME™ program? Please describe. ______________________________

Which of the following conditions cause the balance/mobility limitations of people that register in your TIME™ program?

	Yes	No
15. Stroke	○	○
16. Acquired brain injury	○	○
17. Multiple sclerosis	○	○
18. Parkinson's disease	○	○
19. Other:	○	○

If Other, please specify: ______________________________

20. Typically, what percentage of participants in a TIME™ class are returning participants?

- 0% of participants are returning
- 1-25% of participants are returning
- 26-50% of participants are returning
- 51-75% of participants are returning
- 76-100% of participants are returning

Comments: _______________________________

21. Typically, what percentage of TIME™ participants would like to return, but are unable to re-register because of space constraints?

- 0% are unable to re-register
- 1-25% are unable to re-register
- 26-50% are unable to re-register
- 51-75% are unable to re-register
- 76-100% are unable to re-register

Comments: _______________________________

22. Do you have a waiting list for the TIME™ program?

- Yes

  If Yes, please specify the number of people currently on the waiting list: ____________
- No

Comments: _______________________________

TIME™ Program Delivery

23. What version of the TIME™ exercise program are you currently using?

- Earlier 9-station version
- Modified 3-superstation version
- Other

If Other, please specify: _______________________________

24. How many classes per week are offered in your TIME™ program?

- 1 class per week
- 2 classes per week
- Other

If Other, please specify: _______________________________

25. What is the duration (in minutes) of a single TIME™ class?

- 60 minutes

- ○ Other

If Other, please specify: ______________________________

26. For how many weeks are TIME™ classes offered in one session?

- ○ 4 weeks
- ○ 6 weeks
- ○ 8 weeks
- ○ 10 weeks
- ○ 11 weeks
- ○ 12 weeks
- ○ Other

If Other, please specify: ______________________________

27. How many TIME™ sessions are offered in one year at your community centre? Indicate the total number of sessions you run per year including concurrent sessions. If you are completing the questionnaire for multiple sites, list the site and number of sessions per year in the text box under "Other" (eg Centre 1 name: 6 sessions, Centre 2 name: 4 sessions, etc).

- ○ 2 sessions per year
- ○ 3 sessions per year
- ○ 4 sessions per year
- ○ 5 sessions per year
- ○ 6 sessions per year
- ○ 7 sessions per year
- ○ Other

If Other, please specify: ______________________________

28. What is the typical number of participants in one class? ____________

29. What is the maximum number of participants permitted in one class? ____________

30. What is the minimum number of participants that need to register in order for you to run a class? ____________

31. How many instructors are typically used to run a class?

- ○ 1 instructor per class
- ○ 2 instructors per class
- ○ 3 instructors per class
- ○ Other, please specify...________________________

32. Do you allow volunteers to assist during a TIME™ class?

- ◌ Yes
- ◌ No

33. What is the typical instructor to participant ratio (including volunteers) in your program? E.g., 1 instructor/volunteer to 5 participants
____

34. How many volunteers typically help out in a TIME™ class?

- ◌ 0 volunteers per class
- ◌ 1 volunteer per class
- ◌ 2 volunteers per class
- ◌ 3 volunteers per class
- ◌ Other

If Other, please specify: ________________________________

35. Do you allow caregivers to assist during a TIME™ class?

- ◌ Yes
- ◌ No

36. How many caregivers typically assist in a TIME™ class?

- ◌ 0 caregivers per class
- ◌ 1 caregiver per class
- ◌ 2 caregivers per class
- ◌ Other

If Other, please specify: ________________________________

37. Typically, what percentage of TIME™ participants register for other exercise classes at your centre?

- ◌ 0% register for other classes
- ◌ 1-25% register for other classes
- ◌ 26-50% register for other classes
- ◌ 51-75% register for other classes
- ◌ 76-100% register for other classes

38. If TIME™ participants do register for other classes, please describe the classes to which they progress: _____________

You have completed the survey questionnaire! We sincerely appreciate your time and effort. We will provide everyone with a summary of the results.
