# Supplementary material for: Considerations for expanding community exercise programs incorporating a healthcare-recreation partnership for people with balance and mobility limitations: a mixed methods evaluation
Source: BMC Res Notes. 2018 Apr 2;11:214. doi: 10.1186/s13104-018-3313-x (PMC5879753; doi:10.1186/s13104-018-3313-x)
Supplement: Supplementary file 4 — Additional file 4. Meeting participant positions and organizations. [file 13104_2018_3313_MOESM4_ESM.pdf]

Additional File 4

List of Meeting Participants

**Province**	**City**	**Position**	**Organisation**
British Columbia	Vancouver	Clinical Supervisor	British Columbia Fraser Health Authority
British Columbia	Vancouver	Interim Programme Director	British Columbia Fraser Health Authority
Manitoba	Winnipeg	Assistant Professor	University of Manitoba
Newfoundland	St. John's	Assistant Professor; Clinical Research Scientist, Adjunct Professor; Co-Director Rehabilitation Research Unit of Newfoundland and Labrador	Memorial University
Nova Scotia	Halifax	Physical Therapist, Clinical Specialist (Neuroscience) Interprofessional Clinical Leader for Acquired Brain Injury Services	Nova Scotia Rehabilitation Centre
Ontario	Aurora	Certified Group Fitness Instructor and TIME^{TM} Instructor	Aurora Family Leisure Complex
Ontario	Barrie	Wellness Coach Fitness instructor	Barrie YMCA of Simcoe/Muskoka
Ontario	Barrie	Vice-President of Health & Wellness	Barrie YMCA of Simcoe/Muskoka
Ontario	Barrie	Ontario Stroke Network Regional Community and Long Term Care Coordinator	Central East Stroke Network, Royal Victoria Regional Health Centre
Ontario	Bradford	Former Fitness Supervisor	Bradford West Gwillimbury Leisure Centre
Ontario	Bradford	Fitness Programmer	Bradford West Gwillimbury Leisure Centre
Ontario	Collingwood	TIME^{TM} Program Lead	Collingwood YMCA of Simcoe/Muskoka
Ontario	Gravenhurst	General Manager	Gravenhurst YMCA of Simcoe/Muskoka
Ontario	Gravenhurst	TIME^{TM} Volunteer	Gravenhurst YMCA of Simcoe/Muskoka
Ontario	Gravenhurst	Supervisor, Adult Fitness and Individual Conditioning	Gravenhurst and Orillia YMCA of Simcoe/Muskoka
Ontario	Hamilton	Assistant Professor	McMaster University

Province	City	Position	Organisation
Ontario	Markham	Project Coordinator	Central Local Health Integration Network
Ontario	Orillia	Physical Therapist	Orillia Soldiers' Memorial Hospital
Ontario	Richmond Hill	Community Liaison, District Stroke Centre	Mackenzie Health Richmond Hill Hospital
Ontario	Richmond Hill	Coordinator, District Stroke Centre	Mackenzie Health Richmond Hill Hospital
Ontario	Scarborough	Coordinator, Rehabilitation to Community and Education	Variety Village
Ontario	Stouffville	Fitness Supervisor	Whitchurch-Stouffville Leisure Centre
Ontario	Sudbury	Regional Coordinator	ICAN Independence Centre and Network and Health Sciences North
Ontario	Thunder Bay	Ontario Stroke Network Regional Rehabilitation Coordinator	North Western Ontario Regional Stroke Network
Ontario	Toronto	Facilitator	University of Toronto, Department of Physical Therapy
Ontario	Toronto	Medical Director Brain and Spinal Cord Program	University Health Network - Toronto Rehabilitation Institute
Ontario	Toronto	Advocate for People with Disabilities	City of Toronto Parks Forestry and Recreation, Etobicoke Civic Centre
Ontario	Toronto	Clinical Educator, Physiotherapy	University Health Network - Toronto Rehabilitation Institute
Ontario	Toronto	Community Recreation Programmer: Active Living	City of Toronto Metro Hall Fitness Centre
Ontario	Toronto	Community Recreation Programmer: Active Living	City of Toronto Mary McCormick Recreation Centre
Ontario	Toronto	TIME™ Program Instructor and Coordinator	Harbourfront Community Centre
Ontario	Toronto	Clinical Educator, Physiotherapy	University Health Network - Toronto Rehabilitation Institute
Ontario	Toronto	Senior Manager, Business Management	University Health Network - Toronto Rehabilitation Institute

**Province**	**City**	**Position**	**Organisation**
Ontario	Toronto	Fitness Instructor	City of Toronto Wallace Emerson Community Centre
Ontario	Toronto	Assistant Executive Director	Harbourfront Community Centre
Ontario	Toronto	Stroke Rehab and Community Re-engagement Coordinator	Toronto Western Hospital
Ontario	Toronto	MSc Student	University of Toronto, Rehabilitation Sciences Institute
Ontario	Toronto	Physical Therapist	University Health Network-Toronto Rehabilitation Institute
Ontario	Toronto	Associate Professor	University of Toronto, Department of Physical Therapy
Ontario	Toronto	Director of Geriatrics	University Health Network - Mount Sinai Hospitals
Ontario	Toronto	Ontario Stroke Network Regional Community & Long Term Care Coordinator, West Greater Toronto Area Stroke Network	Trillium Health Partners, Queensway Health Centre
Ontario	Wasaga Beach	General Manager	Wasaga Beach YMCA of Simcoe/Muskoka
Ontario	Wasaga Beach	Personal Trainer/Wellness Coach	Wasaga Beach YMCA of Simcoe/Muskoka
Ontario	Wasaga Beach	Wellness Coach	Wasaga Beach YMCA of Simcoe/Muskoka
Ontario	Whitby	Lead, Health and Wellness Transition	Abilities Centre
Quebec	Montreal	Chef d'administration de programmes - responsable pour qualité, performance, enseignement et recherche dans la direction réadaptation CSSS Cavendish	Cavendish Health & Social Services Centre
Quebec	Montreal	Physical Therapist	Cavendish Health & Social Services Centre
