# Supplementary material for: Considerations for expanding community exercise programs incorporating a healthcare-recreation partnership for people with balance and mobility limitations: a mixed methods evaluation
Source: BMC Res Notes. 2018 Apr 2;11:214. doi: 10.1186/s13104-018-3313-x (PMC5879753; doi:10.1186/s13104-018-3313-x)
Supplement: Supplementary file 5 — Additional file 5. Identification and endorsement of challenges. [file 13104_2018_3313_MOESM5_ESM.pdf]

Additional File 5

Identification and endorsement of challenges to implementing community exercise programs for people with balance and mobility limitations by 40 meeting participants

**Stakeholder Group Identifying Challenge**[*]	**Top Challenges Identified**	**Number of Meeting Participants Endorsing Challenge by Confidential Vote (n)**						**Total Vote**[†] **n (%)**
		**HCP (n=7)**	**HCS (n=9)**	**FI (n=11)**	**RC/M (n=9)**	**Res (n=3)**	**TP (n=1)**	
Healthcare professionals (HCP) (n=7)	1) Lack of available evidence-based exercise programs (e.g., TIME™ program) across province, maintenance of program integrity	6	3	1	2	0	0	**12 (30)**
	2) Lack of availability of a range of exercise programs that target low to high function	1	0	0	0	0	0	1 (3)
Healthcare system (HCS) (n=9)	3) Selection of fitness instructors with the appropriate skill set	0	0	2	0	0	0	2 (5)
	4) Sustainability (of healthcare-recreation partnerships): funding, role maintenance, ensuring maintenance of community engagement	0	5	1	3	1	0	**10 (25)**
	5) Integration and ensuring multiple agencies are aware of what the other is doing; marketing	0	1	0	0	0	0	1 (3)
Fitness instructors (FI) (n=11)	6) Staff training: training instructors to have multiple skills required to deliver the exercise programs (e.g., adapting exercise difficulty to account for participant ability or injury)	0	0	5	2	0	0	7 (18)
	7) Program promotion and awareness (e.g., among rehabilitation and recreation providers, doctors, healthcare teams/centres)	1	2	6	3	0	0	**12 (30)**
	8) Low participant registration: no access to rehabilitation programs; need for other ways to identify participants	0	0	0	0	0	0	0 (0)
Recreation coordinators /managers (RC/M) (n=9)	9) Cost recovery, sustainability, expansion (e.g., equipment, wages)	0	0	1	6	2	0	**9 (23)**
	10) Participant recruitment is either too low or too high at different sites	0	0	2	2	0	0	4 (10)
Researchers (Res) (n=3)	11) Insufficient funding for the recreation provider to run the exercise program	1	1	0	0	0	0	2 (5)
	12) Insufficient funding for the healthcare partner to offer training and support for the exercise program	1	2	1	0	3	0	7 (18)
TIME™ participant (TP) (n=1)	13) Transportation from home to community centre is expensive (e.g., $10 one way)	2	4	1	1	0	1	**9 (23)**
	14) Transportation schedule is inconvenient (e.g., adapted transport arrives 45 min early)	0	4	1	1	0	1	7 (18)
Various	15) Volunteers[‡] (training and scheduling)	0	0	3	2	0	0	5 (13)

[*]Each stakeholder group was seated at a separate table; fitness instructors were seated at two tables.
[†]Bolded results indicate challenges endorsed by ≥20% of meeting participants.
[‡]New challenge participants identified during anonymous voting. Some challenges were combined and addressed in the prioritization stage.
